# Engineering Functional Particles to Modulate T Cell Responses

**DOI:** 10.1021/accountsmr.4c00105

**Published:** 2024-07-18

**Authors:** Yudong Li, Shukun Li, Jari F. Scheerstra, Tania Patiño, Jan C. M. van Hest, Loai K. E. A. Abdelmohsen

**Affiliations:** † Bio-Organic Chemistry, Institute for Complex Molecular Systems, Eindhoven University of Technology, 5600 MB Eindhoven, The Netherlands; § State Key Laboratory of Biochemical Engineering, Institute of Process Engineering, Beijing 100190, China

## Abstract

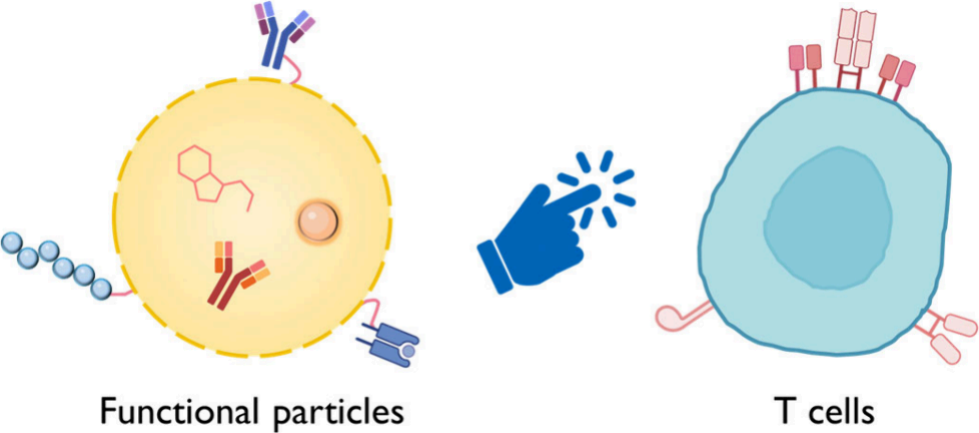

T cells play a critical role in adaptive immune responses. They work with other immune cells such as B cells to protect our bodies when the first line of defense, the innate immune system, is overcome by certain infectious diseases or cancers. Studying and regulating the responses of T cells, such as activation, proliferation, and differentiation, helps us understand not only their behavior *in vivo* but also their translation and application in the field of immunotherapy, such as adoptive T cell therapy and immune checkpoint therapy, the situations in which T cells cannot fight cancer alone and require external engineering regulation to help them. Nano- to micrometer-sized particulate biomaterials have achieved great progress in the assistance of T cell-based immunomodulation. For example, various types of microparticles decorated with T cell recognition and activation signals to mimic native antigen-presenting cells have shown successful *ex vivo* expansion of primary T cells and have been approved for clinical use in adoptive T cell therapy. Functional particles can also serve as vehicles for transporting cargos including small molecule drugs, cytokines, and antibodies. Especially for cargos with limited bioavailability and high repeat-dose toxicity, systemic administration in their free form is difficult. By using particle-assisted systems, the delivery can be tailored on demand, of which targeting and controlled release are two typical examples, ultimately aiding in the regulation of T cell responses. Furthermore, when T cells become overactive and behave in ways that contradict our expectations, such as attacking our own cells or innocuous foreign molecules, this can lead to a breakdown of immune tolerance. In such cases, particles to help reprogram those overactive T cells or suppress their activity are appreciated *in vivo*. The urgent need to introduce immune stimulation into the treatment of cancers, infectious diseases, and autoimmune diseases has driven recent advances in the engineering of functional particulate biomaterials that regulate T cell responses. In this Account, we will first cover a brief overview of the process of T cell-based immunomodulation from principle to development. It then outlines critical points in the design of functional particle platforms, including materials, size, morphology, surface engineering, and delivery of cargos, to modulate the features of T cells, and introduces selected work from our and other research groups with a focus on three major therapeutic applications: adoptive T cell therapy, immune checkpoint therapy, and immune tolerance restoration. Current challenges and future opportunities are also discussed.

## 1 Introduction

**Figure 1:**
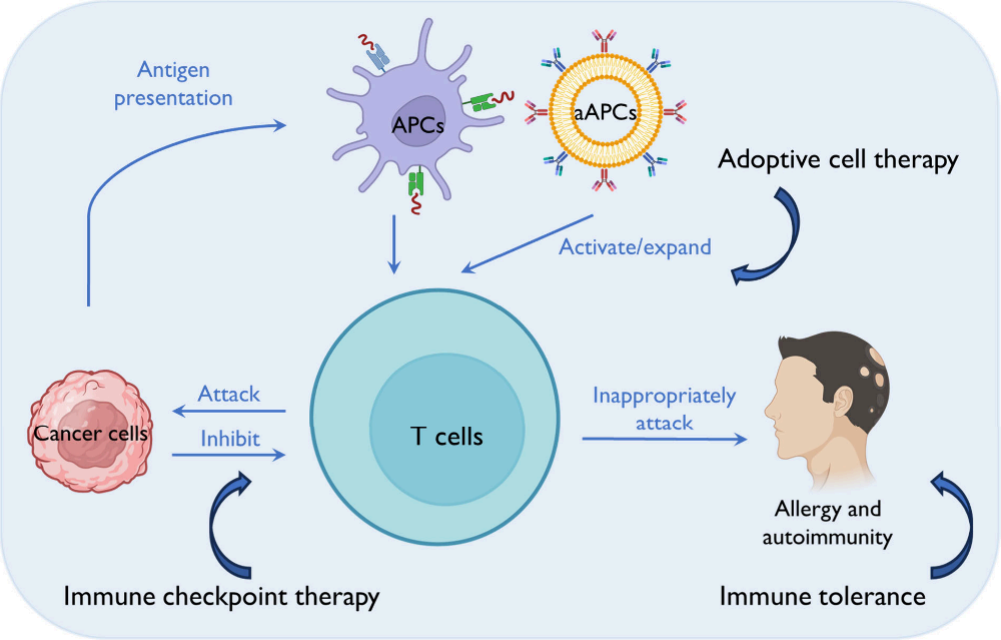
Overview of T cell-based immunomodulation.

Adaptive immunity, also known as acquired immunity, is a protective mechanism mediated by B cells and T cells that is highly specific against particular infectious agents encountered by the body. Its main characteristic is that after an initial response to a specific pathogen, an immune memory is created, and a stronger response is generated in future encounters with that pathogen. In many cases, when our bodies are unable to fight these pathogens, such as a wide variety of viruses and bacteria, we may need to introduce some engineering tools and approaches to help the immune system become “more active”. Cancer immunotherapy is a prime example of boosting immunity in this regard. These therapeutics have grown tremendously over the past few decades, thanks to the continued advancement of novel and integrative therapies, such as adoptive T cell therapy (ACT) and immune checkpoint therapy (ICT), which complement or replace traditional surgery, chemotherapy, and radiotherapy.^1^ In addition, the COVID-19 pandemic over the past four years has witnessed and accelerated the development and application of immunomodulatory methods, particularly nanoparticle-based vaccines. The most well-known example is the messenger RNA (mRNA) vaccines, which have an enhanced ability to elicit robust T cell responses and to protect against severe disease.^2^ This achievement was also recently recognized by the 2023 Nobel Prize in Physiology or Medicine. Moreover, self/nonself recognition is a critical aspect of adaptive immunity. At times, immune cells become overactive, leading them to mistakenly attack our own cells or innocuous foreign molecules, resulting in a breakdown of immune tolerance. For instance, when a patient undergoes an exogenous organ transplant, medications to suppress the immune system are often necessary to reduce the risk of rejection. Additionally, considerable attention has been devoted to understanding the dysregulation of autoimmunity, encompassing conditions such as alopecia areata, systemic lupus erythematosus, and rheumatoid arthritis. In such scenarios, the objective is to attenuate the immune system’s activity through external regulation. In this context, it is of great significance to review and summarize the most recent examples of engineering to modulate immune responses. While it is challenging to specifically discuss one type among the various types of immune cells, as they consistently interact with each other, the focus of this Account is on the direct modulation of T cell responses via biomaterial engineering. Furthermore, since macroscale materials such as scaffolds, which are also important in this regard, have been discussed elsewhere,^3^ we have narrowed our scope down to nano- to micrometer-sized particles (NPs and MPs), an area in which our group has extensive experience. In this Account, we first examine the regulation of T cells and explore how these regulations contribute to T cell-based immunomodulation (Figure 1). Key principles of particle design and their application in regulating T cell behaviors are highlighted. Finally, challenges and future opportunities are discussed. This Account can serve as a toolbox for material scientists and immunologists to develop engineering strategies for T cell-based immunomodulation.

## 2 Overview of T Cell-Based Immunomodulation

While a comprehensive introduction to the adaptive immune system cannot be provided here, we give a brief overview of the function of T cells in immunity and their use in immunotherapy to help the reader better understand the following sections. Additionally, we would like to recommend more in-depth and excellent reviews for those interested.^1,4^

It has become clear that adaptive immunity is controlled by and also provides feedback to innate immunity. Innate immunity is the first line of defense of the immune system and involves macrophages, among others. These cells directly recognize pathogenic and diseased states in the body via pattern-recognition receptors (PRRs) which interact with conserved motifs such as pathogen-associated molecular patterns (PAMPs) or damage-associated molecular patterns (DAMPs), leading to an inflammatory response. However, the innate immune response can be overcome by certain infections. In such cases, naïve antigen-presenting cells (APCs) such as dendritic cells (DCs) are activated. They digest antigens from pathogens or self-proteins and present them as short peptides on major histocompatibility complex I or II (MHC-I or MHC-II). Subsequently, APCs exhibiting these characteristics migrate to lymph nodes and activate naïve T cells.

T cells, also known as T lymphocytes, are a type of white blood cells generally classified into two populations: CD4^+^ T cells and CD8^+^ T cells. CD8^+^ cells, also referred to as cytotoxic T cells (CTLs), directly carry out killing functions that are “toxic” to other cells, such as destroying cells infected by pathogens, tumor cells, or otherwise damaged cells. CD4^+^ T cells can be categorized into T helper (T_h_) cells and regulatory T (T_reg_) cells. T helper type 1 (T_h1_) cells do not kill cells directly but secrete cytokines including Interleukin-2 (IL-2) and Interferon-gamma (IFN-γ), which promote the activation of macrophages and CTLs to kill infected cells. T helper type 2 (T_h2_) cells communicate with B cells via the production of cytokines, such as IL-4, IL-5, and IL-13, activating them to become plasma cells capable of secreting antibodies. T_h17_ cells mediate the host’s defense mechanism against microbe infections, such as extracellular bacteria and fungi, complementing T_h1_ and T_h2_ immunity. T_h17_ cells are also involved in immune tolerance. T_reg_ cells act as a counterbalance, reducing the activity of other T cells when needed. Under normal circumstances, they suppress or prevent inappropriate immune responses such as T cells attacking healthy cells, a condition known as autoimmune disease.

Due to the critical role T cells play in the adaptive immune system, research on T cell behaviors and functions has attracted great attention since the last century. For example, many studies have demonstrated how to regulate T cells *in vitro*/*ex vivo*. Not only has this helped us better understand how this process works in the body, but many of the findings have also been successfully translated into breakthroughs in clinical therapies such as ACT. ACT is a cancer treatment that uses a patient’s own T cells with antitumor activity, expanding them *ex vivo* before infusing them back into the patient.^5^ A good example is chimeric antigen receptor (CAR) T cell therapy, the first gene transfer therapy approved by the U.S. Food and Drug Administration (FDA).^6^ It strengthens the patient’s own T cells by inserting genes for CAR, thereby improving the specificity and ability of our immune system to kill cancer cells. As with other ACT therapies, the engineered T cells should be ideally expanded *ex vivo*. Given the cumbersome and lengthy process of extracting and utilizing autologous DCs, biomaterials that mimic the features of DCs for the activation and expansion of T cells have been developed and named artificial antigen-presenting cells (aAPCs).

T cell activation generally requires the combination of signals from T cell receptors, coreceptors, and costimulatory receptors (Figure S1). Cytokines can further determine the T cell responses, including proliferation and differentiation. An introduction to the signals required for T cell activation is provided in the Supporting Information.

Meanwhile, there are mechanisms in the body to maintain immune homeostasis such as preventing self-attack against own molecules, known as immune tolerance. T_reg_ cells play an essential role in this process through inhibitory cytokine secretion and direct contact suppression.^7^ However, when tolerance is broken and a process similar to normal T cell activation occurs with self-antigens or innocuous foreign antigens instead of pathogens, conditions such as autoimmunity, allergies, and transplanted organ rejection can occur. In these cases, immunomodulation is needed to restore the balance of T cell function. Speaking of self-tolerance, there are negative pathways regulated by immune checkpoints, which prevent indiscriminate self-attack when functional. Programmed cell death 1 (PD-1) and cytotoxic T lymphocyte antigen 4 (CTLA-4) are the most well-known immune checkpoints on T cells. Some tumor cells have exploited this by developing partner proteins, such as programmed death-ligand 1 (PD-L1), that can bind to checkpoints to evade attack. Correspondingly, a promising class of T cell-based cancer immunotherapies, ICT, has been elaborated to block one or multiple receptors using antibodies, thereby reversing the “off” signals induced by tumor cells and reinvigorating the functions of T cells. This revolutionary discovery received the Nobel Prize in Physiology or Medicine in 2018. Excellent reviews on this topic, from principles to development to clinical advances, can be found here.^8−10^

In the following section, we will discuss the principles of modulating T cell behavior and function by tuning the design of particulate biomaterials.

## 3 Design of Functional Particles for T Cell-Based Immunomodulation

### 3.1 Materials

**Figure 2:**
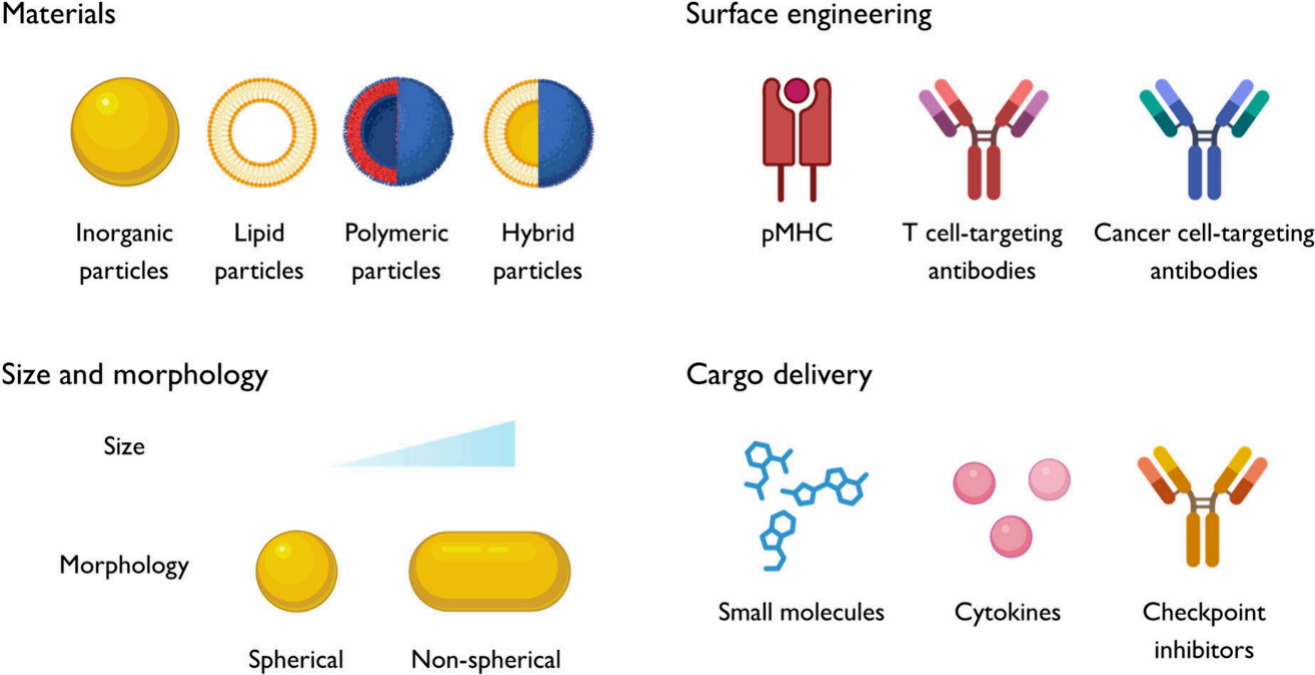
Overview of particle design for T cell-based immunomodulation.

Generally, a variety of NPs and MPs, including inorganic particles, lipid particles, and polymeric particles, have been used for T cell modulation (Figure 2).^11^ The choice of materials, along with preparation and processing methods, directly affects the architecture and properties of the assembled particles, thereby playing a pivotal role in the regulatory process. In addition to size and morphology, arguably two of the most discussed factors in the literature, these properties include particle stability, compatibility, membrane fluidity, and stiffness. When it comes to the manufacture of aAPCs for T cell expansion *ex vivo*, the first example that comes to mind is Dynabeads functionalized with anti-CD3 and anti-CD28, which have been utilized as the “gold standard” for the high-throughput production of clinical-grade T cells.^12^ These beads are typically polystyrene beads, encapsulated with superparamagnetic Fe_3_O_4_ NPs. They are easily handled and separated by magnets during use without compromising their biocompatibility, as the shell protects cells from exposure to Fe_3_O_4_. Previous studies have shown that these beads successfully shorten the time-consuming expansion process from over 40 days to around 10 days with ideal expansion efficiency of more than 200-fold. This efficiency can be further optimized by varying the ratio of beads to cells or the ratio of anti-CD3 to anti-CD28.^13^ Dynabeads are now commercially available and have given rise to various types of products. For instance, additional anti-CD137 signaling has been introduced to promote the survival and proliferation of T cells. The standardized products offer great potential to improve reproducibility in research and clinical applications.

**Figure 3:**
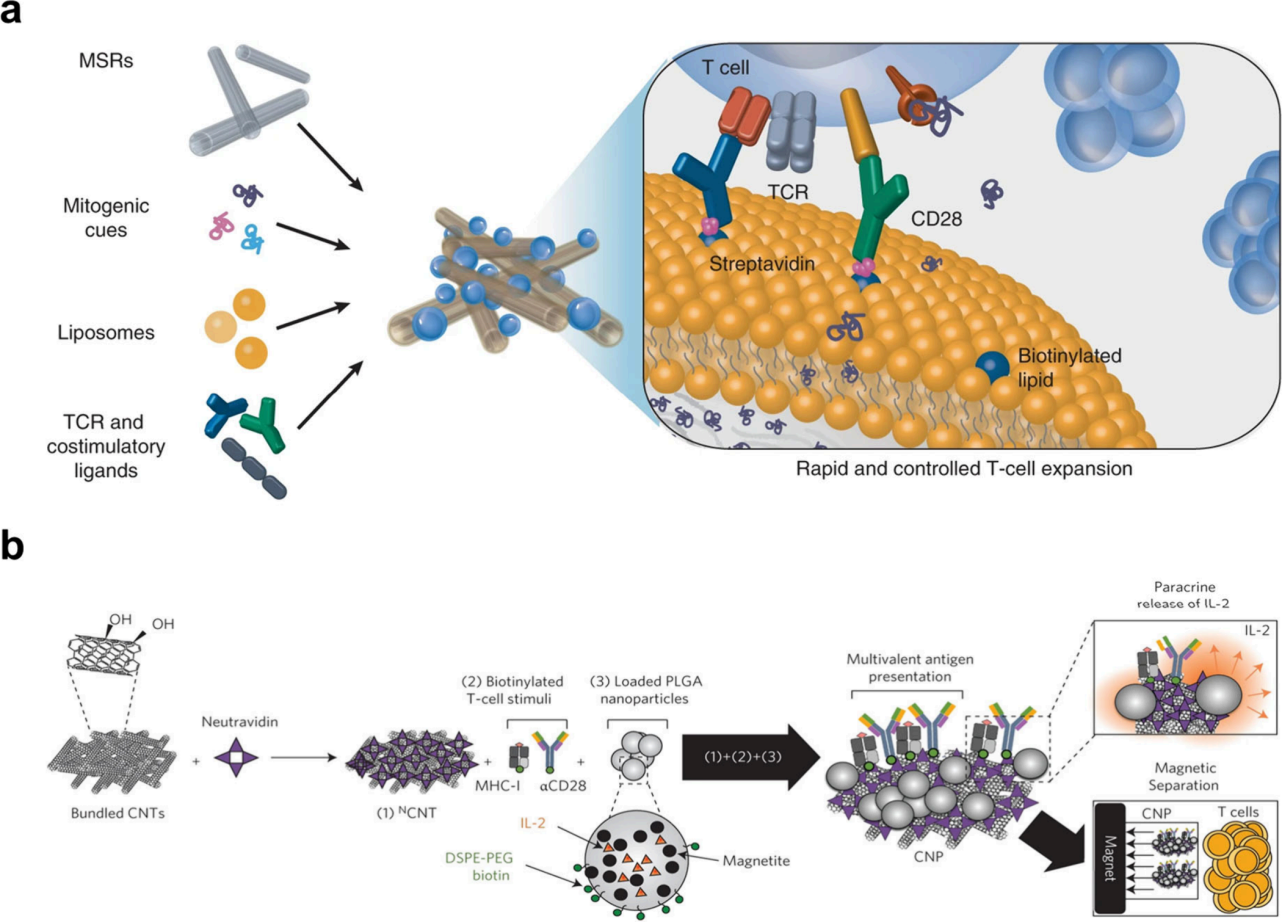
Hybrid particle systems designed for T cell activation and expansion. (**a**) Schematic illustration of liposome-coated mesoporous silica microrods (MSRs). Reproduced with permission from ref (17). Copyright 2020 Springer Nature. (**b**) PLGA NP-loaded carbon nanotubes (CNTs). Reproduced with permission from ref (18). Copyright 2014 Springer Nature.

In contrast to rigid MPs represented by Dynabeads, which lack dynamics, liposomes, composed of lipid bilayers, have also been used to constitute aAPCs.^14,15^ The key feature of biomimetic liposome-based aAPCs is that the lipid bilayers are considered to be dynamic, providing ideal membrane fluidity during interaction with cells. This mobility also facilitates the reshaping of vesicle structures and the reorganization of ligands on the membrane. Despite the advantages, liposome-based aAPCs have limitations from preparation to application, including a limited number of available lipids, low particle stability during storage, and insufficient stiffness to provide the necessary mechanical signals for T cell stimulation. As a result, studies based purely on liposomes have gradually fallen out of focus. As an alternative, lipid-coated rigid particles, which combine the advantages of particle stiffness and membrane fluidity control, have attracted considerable attention. For example, by decorating mesoporous silica microrods (MSRs) with a fluid lipid (POPC) bilayer, the Mooney group developed a type of aAPCs with three signals required for T cell activation, inducing T cell expansion 2–10 times more effectively than Dynabeads (Figure 3a).^16,17^ To demonstrate the promising potential of the MSRs in immunotherapy, the authors also showed that MSRs were five times more effective at expanding CD19-specific CAR T cells than Dynabeads.

Besides small molecule lipid-based particles, polymer-based systems have been extensively studied. Compared to lipids, polymers can be easily customized on demand, which increases the potential for large-scale preparation of particles for cellular applications. For example, functional groups such as carboxylic acid, amine, azide, and alkyne groups can be introduced into the polymer, allowing for convenient postpolymerization functionalization or postassembly modification.

In addition to the polystyrene platform on which Dynabeads are based, several other biologically derived and synthetic polymeric particles have been used for T cell modulation. For instance, for T cell differentiation, Butte and co-workers prepared cytokine-loaded monodispersed MPs based on alginate and heparin, two naturally occurring biocompatible polysaccharides.^19^ Additionally, through a combination of microfluidics and gelation, various particle and mesh sizes can be easily tuned, providing great control over particle properties. Another well-studied polymeric system is based on biodegradable poly(lactic-*co*-glycolic acid) (PLGA), which has been approved by the U.S. FDA and the European Medicines Agency (EMA) for preclinical and clinical studies.^20^ By presenting the three signals required for T cell stimulation, Fahmy and Steenblock developed PLGA-based aAPCs that allowed sustained release of IL-2, resulting in a 45-fold enhancement of T cell expansion, and this mode of expansion is biased toward the CD8^+^ T cell phenotype.^21^ Similar to lipid-coated systems, researchers have also created aAPCs for T cell expansion *ex vivo* by combining polymer materials with inorganic materials. Fahmy and co-workers first conjugated antigens to carbon nanotubes, encapsulated IL-2 and magnetite in PLGA NPs, before combining the two entities (Figure 3b).^18^ The results showed that the nanotube–polymer composite expanded CD8^+^ T cells *ex vivo* to a level comparable to clinical standards using a thousand-fold less soluble IL-2. In a murine melanoma model, injection of the expanded lymphocytes directly into tumors was shown to significantly delay tumor growth. Figdor and co-workers coated SIINFEKL-bound MHC and anti-CD28-conjugated semiflexible poly(isocyanopeptide) immunofilaments on magnetic beads to mimic a fluidic membrane, strongly amplifying and enriching antigen-specific T cells by more than 90-fold in 7 days.^22^

DNA scaffolds have recently emerged as a new class of materials for T cell regulation. Precise spatial control of ligand presentation on aAPCs can be exploited to engineer ligand–receptor interactions and allow for quantitative analysis at single-particle resolution.^23^ DNA has also been conjugated to biodegradable polymers and then assembled into particles. The scaffolds on the particle surface enable efficient loading of complementary DNA–biomolecule complexes based on direct hybridization. This versatile platform has shown great potential in achieving T cell expansion and local activation of AND-gate CAR-T cells.^24^

From our perspective, hybrid particles, such as MSRs and CNTs, are the most promising platforms that perform multiple functions by combining various types of components, masking the drawbacks of a single material.

### 3.2 Size and Morphology

Almost all of the samples discussed above for T cell modulation *ex vivo* are MPs. In early studies, scientists noticed that size had a significant impact on T cell responses. As for activation, latex MPs with a size of 4–5 μm were optimal, while the responses decreased rapidly with decreasing particle size, and even an increase in particle number could not compensate for the suboptimal size.^25^ This benefit from the large size effect can be attributed to the fact that large particles can provide a large and continuous surface contact area for T cell stimulation through mechanical cues or the mobilization of more ligands. However, when these MPs are used in the body, their administration pathways and applications are restrained because they have limited vascular and lymphatic drainage and are easily engulfed by phagocytes. Therefore, particles at the nanoscale are preferred for T cell modulation *in vivo*. They can be administered into the body in various ways, including intravenous injection, and are designed to target different organs and tissues as needed.^26^ The sequential differences in delivery and targeting behavior are also the most obvious distinction between nanoparticles and macroscale materials such as hydrogels, which are better suited to be placed at the targeted site for *in situ* delivery of immunomodulators, with the possibility of subsequent entry into the systemic circulation.^3^ For nanoparticles, their size affects the targeting behavior, including biodistribution, ability to penetrate barriers, as well as cellular uptake and clearance, which determines whether they reach the desired location.^26^ In addition, size directly impacts T cell activation and expansion.

Schneck and colleagues studied the size effect of aAPCs based on well-defined superparamagnetic iron oxide NPs.^27^ In this regard, NPs with diameters of 50, 300, 600, and 4500 nm, each carrying two signals (pMHC and anti-CD28), were achieved. The results showed that, when normalizing all the groups by the total number of pMHCs bound, aAPCs larger than 300 nm could activate CD8^+^ T cells more efficiently (approximately 2.4-fold) than 50 nm ones, while the latter could only activate the T cells at saturating doses or when forming clusters induced by a magnetic field. This difference may be attributed to the fact that the local island formation of the two signals is larger than 50 nm for effective T cell stimulation.

Nonspherical particles, including ellipsoidal and tubular particles, with high aspect ratios (ARs) are of growing interest in cellular applications. After *in vivo* administration, shape modulates the fate of particles by affecting their transport and biodistribution.^28^ Furthermore, it has been shown that antibody-coated rods reduce nonspecific cellular uptake compared to spheres.^29^ As for T cell modulation, morphology is also identified as a key parameter that can be engineered to manipulate the interaction between particles and cells. Similar to size, the design consideration for the elongated structure of particles is to increase the contact area between the long axis of the particle and the cell, thereby directly affecting cell function. In addition, this facilitates the formation of cell clusters around the particle, as demonstrated by MSRs and CNTs mentioned above.^16,18^ Another study on the shape effect was performed using ellipsoidal PLGA aAPCs with varying ARs.^30^ An orderly increase in CD8^+^ T cell expansion behavior was observed with greater stretching of the particles, from spherical aAPCs to ellipsoidal aAPCs. The enhanced activity was also observed *in vivo*, as high-AR aAPCs significantly improved mouse survival in a melanoma model. Similar effects were found when using gold NPs, with high-AR nanorods significantly enhancing T cell expansion, while morphological features associated with T cell maturation were observed.^31^

**Figure 4:**
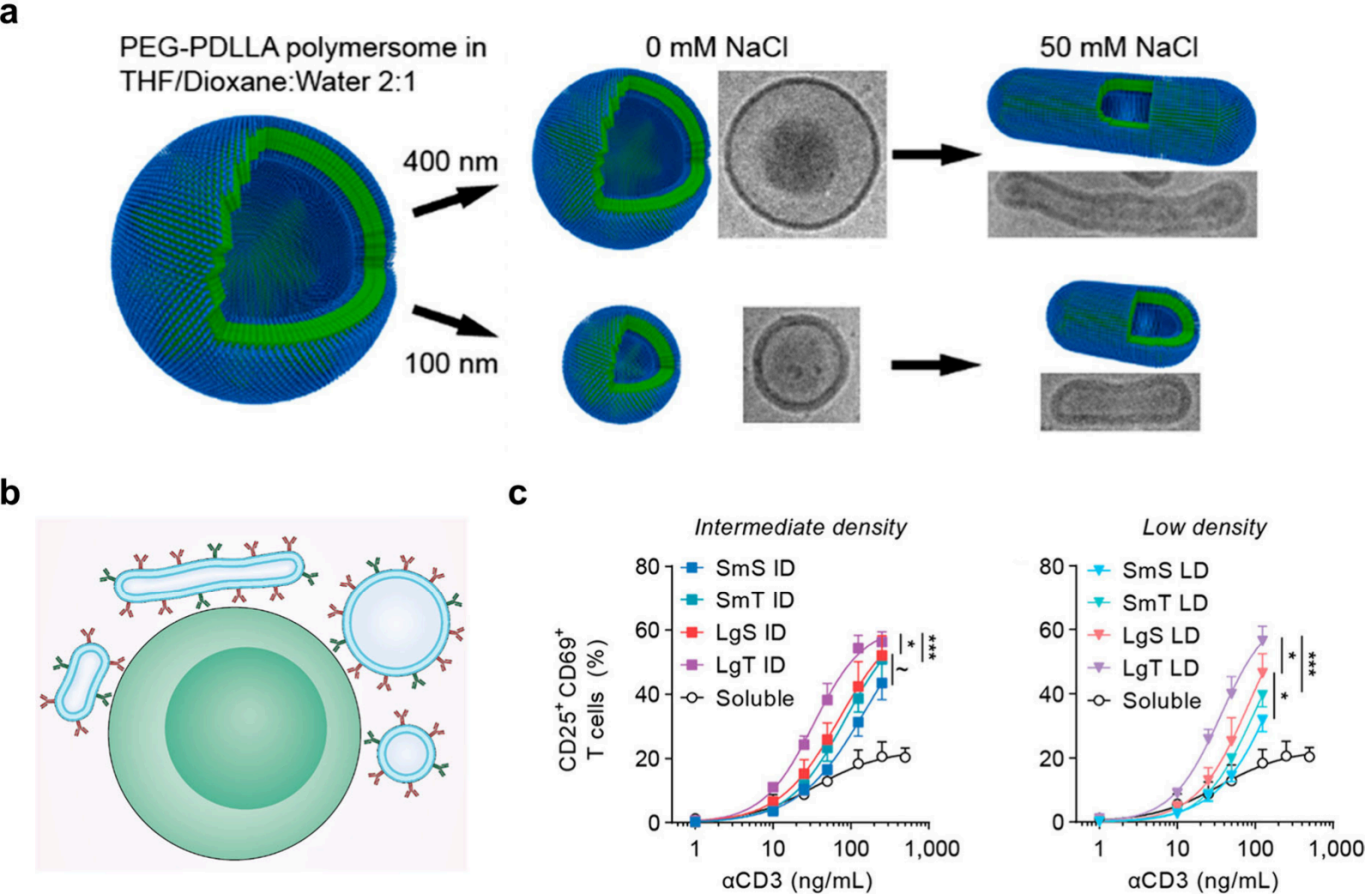
Effect of particle size and morphology on T cell activation. (**a**) Schematic illustration of the extrusion of PEG-PDLLA polymersomes using 400 and 100 nm filters. Their shape transformation under osmotic pressure forms nanotubes. Reproduced with permission from ref (34). Copyright 2018 American Chemical Society. (**b**) Illustration depicting different sizes and shapes of aAPCs based on the polymersomes for T cell modulation. (**c**) Coexpression of CD25 and CD69 measured after 1 day of culture with aAPCs of intermediate antibody density (ID, αCD3/αCD28 spacing: ∼40/30 nm) and low antibody density (LD, αCD3/αCD28 spacing: ∼60/50 nm). SmS = small spheres, SmT = small tubes, LgS = large spheres, LgT = large tubes. Reproduced with permission from ref (35). Copyright 2022 American Chemical Society.

To systematically determine size and shape effects, precise control of particle design is highly appreciated. Our group reports a series of micro- and nanoscale artificial cells constructed based on bottom-up approaches.^32,33^ As an excellent example, we established a robust system to prepare biodegradable poly(ethylene glycol)-*block*-poly(d,l-lactide) (PEG-PDLLA) polymersomes with topology control through extrusion and osmotic pressure-induced shape transformation (Figure 4a).^34^ Further functionalization with distinct densities of anti-CD3 and anti-CD28 created an aAPC library that allowed a comprehensive study to delineate the impact of topology on T cell activation and expansion (Figure 4b).^35^ It was demonstrated that, at low ligand densities of the antibodies, the effects of both the shape and size of polymersomes were more pronounced, with large and elongated polymersomes showing better activation of T cells than their spherical or smaller counterparts (Figure 4c).

In summary, for efficient T cell expansion *ex vivo*, large and nonspherical particles typically show the greatest potential. When used *in vivo*, it is worth noting that a critical point that is less clear than size studies is how well the designed morphology is maintained under complex physiological conditions. Nonetheless, we conclude that future aAPC development for *in vivo* applications needs to meet certain size and morphology requirements. For example, a trade-off between larger size (for stronger T cell stimulation) and smaller size (for certain applications such as direct activation of tumor-infiltrating CD8^+^ T cells) should be considered in the design.

### 3.3 Surface Engineering

Compared to size and morphology, material surface modification and functionalization have a more direct effect on T cells. The introduction of immunologically active molecules, such as antigens, can serve as an effective strategy for engineering particles.

As mentioned, signal 1 and signal 2 are required for optimal T cell activation and expansion. For the development of aAPCs, conjugating commercially available anti-CD3 and anti-CD28 to particles is the most common choice to meet this requirement. The method is straightforward, low-cost, and has been successfully applied to Dynabeads, etc. However, the application of anti-CD3 will cause aAPCs to indiscriminately activate all cells bearing the CD3 protein, which limits their ability to achieve specific activation of cells *in vitro* and essentially makes *in vivo* application risky. To address this problem, pMHC is often implemented to acquire antigen-specific T cell responses. For example, after confirming polyclonal T-cell expansion with antibody-coated MSRs, anti-CD3 was replaced with a MHC class I monomer presenting the antigens SIINFEKL, or CLG and GLC for the expansion of primary mouse T cells and primary human T cells, respectively.^16^ In this way, antigen-specific T cell responses were achieved from purified mouse CD8^+^ T cells or human peripheral blood mononuclear cells (PBMCs). As for costimulatory signals, anti-CD28 still accounts for the majority of aAPC research and clinical applications. As an alternative or in combination with anti-CD28, particle-assisted antibody stimulation to other receptors, such as OX-40 and CD137, has also received attention.^36−38^ Wang et al. developed a combination immunotherapy using anti-OX-40 and an immune checkpoint inhibitor anti-PD-1-coated NPs to generate dual-binding events with T cells. This approach blocks the negative signal pathway and stimulates T cells simultaneously, resulting in better outcomes than free antibodies.^39^

As for autoimmunity, NP intervention can also be facilitated through surface functionalization. Santamaria and colleagues demonstrated a promising case wherein antigen-specific autoreactive T cells can be efficiently reprogrammed to T_reg_ cells using pMHC-coated PEGylated iron oxide NPs in several mouse models.^40,41^ The success can be attributed to the sustained assembly of NP clusters on the interface, which enhances the affinity between antigen and receptor and prolongs the interaction time. This enhancement mirrors the typical augmentation of effector T cell responses through increased ligand density.^35^ In this case, the critical parameter is the density of pMHC, and the reprogramming of cell functions can only be achieved when the density is above a certain threshold.^41^ García et al. investigated the effect of Fas ligand-immobilized microgels on islet transplantation in diabetic mice. Combined with short-term rapamycin treatment, graft acceptance was prolonged (>200 days), with T_reg_ cells playing a dominant role.^42^

T cell targeting moieties can be integrated with tumor targeting moieties to directly form a connection between T cells and tumor cells, which helps T cells exert cytotoxic activity on tumor cells and induce apoptosis. This type of antibody is called a bispecific T cell engager (BiTE).^43^ However, on-target off-tumor toxicity and associated neurotoxicity are key issues for BiTE-assisted immunotherapy. To address this issue, Mitchell et al. developed a switchable bispecific T cell nanoengager (SiTE) by assembling polymer-conjugated Fab fragments of CD3 and HER2 into particles of approximately 60 nm in size, which can induce antitumor activity both *in vitro* and in a humanized immune system mouse model.^44^ This switchable feature means the signal can be turned “off” due to particle disintegration induced by a subsequent infusion of amantadine. A similar concept has also been extended to NP-based platforms by decorating PEGylated liposome NPs with anti-CD3 for T cells and anti-CD20 for cancer cells.^45^

Importantly, T cell immune checkpoint molecules, primarily PD-1 and CTLA-4, can be targeted for NP functionalization to reverse the immunosuppressive microenvironment and potentially enhance therapeutic effects, particularly in eradicating tumor residues and preventing their recurrence and metastasis. For example, Goldberg and colleagues utilized the antibody fragment conjugation method to modify the surface of NPs, enabling them to target PD-1^+^ cells.^46^ By virtue of their targeting property, these NPs could deliver the TGFβ signaling inhibitor to the cells and extend the tumor-bearing mice survival, which was unachievable with their free drug counterparts. Importantly, these NPs could also enable PD-1-targeted delivery of a TLR7/8 agonist into the tumor microenvironment, which not only increased the proportion of tumor-infiltrating CD8^+^ cells, but also sensitized tumors to subsequent anti-PD-1.

In brief, for material surface engineering, the following aspects have a great impact on T cell modulation: 1) intrinsic ligand properties such as affinity; 2) ligand density; and 3) ligand arrangement at the spatiotemporal level, including ligand spacing or mobility. Multivalent and multispecific interactions between particles and T cells can be utilized to better control the level of cellular response on demand.^47^

### 3.4 Cargo Delivery

Delivery of cargos, such as small molecule drugs, cytokines, and antibodies, is another strategy to modulate T cell responses and can be ultimately applied for immunotherapy. Especially for cargos with limited bioavailability and high repeat-dose toxicity, systemic administration in their free form is difficult. Therefore, the use of particles as a carrier that can provide sustained release of drugs represents a solution to these problems, while sometimes providing synergistically enhanced therapeutic benefits.

In the development of aAPCs, cytokines such as IL-2, IL-12, TNF-α, and IFN-γ are often encapsulated in particles and sustained released in the milieu to promote effector T cell expansion.^48^ On the other hand, cytokines such as TGF-β, administered in a similar manner, enhance the stimulation of T_reg_ cells and can be used to treat autoimmune diseases.^48^ In addition, immunosuppressive drugs, such as the mTOR inhibitor rapamycin and therapeutic antibodies, can also contribute to the treatment of autoimmunity. For ICT to reinvigorate the functions of T cells, anti-PD-1 and anti-CTLA-4 are popular options, and their encapsulation in NPs enhances their specificity and reduces off-target toxicity. The selection and encapsulation modes of small molecule drugs, cytokines, and antibodies have been extensively summarized elsewhere.^49,50^ Here, we would like to focus on the controlled release of these cargos and how these contribute to T cell modulation.

**Figure 5:**
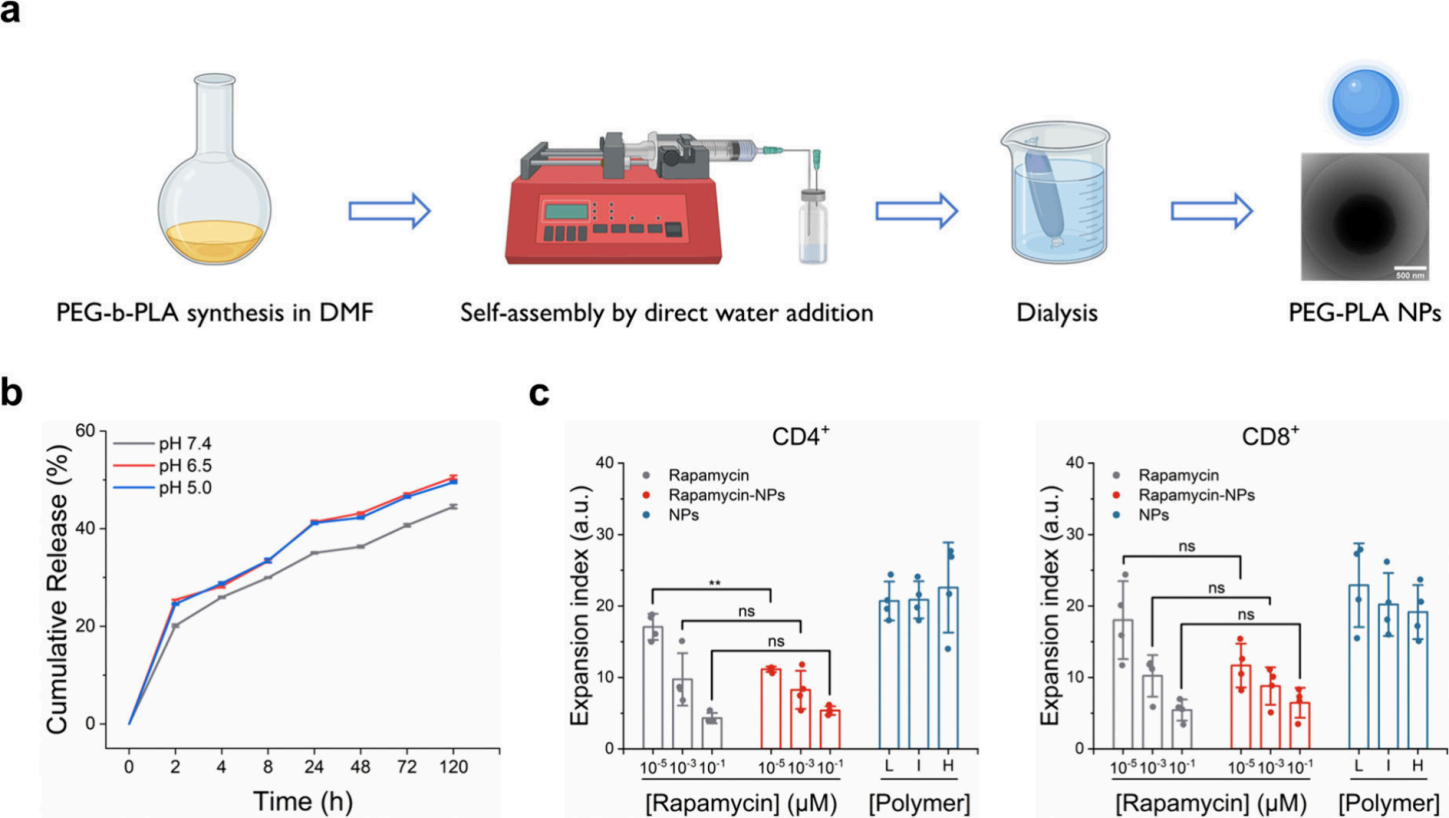
Effect of rapamycin delivery on T cell expansion. (**a**) Schematic illustration of the continuous process for the preparation of PEG-PDLLA NPs. (**b**) Cumulative release of rapamycin from the NPs in PBS buffer (pH 7.4/pH 6.5/pH 5.0) at 37 °C for 120 h. (**c**) Expansion index of CD4^+^ and CD8^+^ T cells determined through flow cytometric analysis after 90 h. The three polymer concentrations in the unloaded NP group corresponded to the three polymer concentrations in the rapamycin-loaded NP group, respectively: H, high concentration; I, intermediate concentration; L, low concentration. ns: *p* > 0.05, **: *p* < 0.01. Reproduced with permission from ref (51). Copyright 2024 John Wiley and Sons.

Our group recently reported a facile method to construct drug-loaded PEG-PDLLA NPs.^51^ In this case, skipping polymer purification before particle assembly greatly facilitates scalable synthesis of polymeric NPs (Figure 5a). Moreover, the micelle-like structure of the NPs resulted in a relatively high encapsulation efficiency (22%) of the hydrophobic agent rapamycin and further restrained the diffusion/permeation of the buffer through the NP matrix, ultimately leading to a well-controlled sustained release (∼50% release after 5 days) (Figure 5b). Moreover, *in vitro* T cell suppression assays showed that rapamycin-loaded NPs exhibited similar or even better inhibitory performance to CD4^+^ and CD8^+^ cells than free rapamycin at different rapamycin concentrations (Figure 5c). This NP matrix degradation-determined release raises our possibility to precisely control the therapeutic window of drugs, and this stability against environmental influences can reduce premature release and enhance the *in vivo* performance of NPs.^52^ In another case, Little et al. demonstrated that a MP system, which slowly releases a T_reg_-recruiting chemokine in a linear fashion, prevented rejection and promoted hindlimb allograft survival (>200 days) in a rat model.^53^

In addition to delivery systems with predetermined release profiles, “smart” delivery systems that can respond to internal or external stimuli represent an alternative strategy. For example, Gu and colleagues designed microneedle-assisted delivery of pH-sensitive NPs encapsulating glucose oxidase (GOx) and anti-PD-1.^54^ The self-dissociation of the NPs can be triggered when GOx converts glucose into gluconic acid, resulting in localized pH decrease, which yields the delivery of anti-PD-1 and prolonged animal survival in a mouse melanoma model. Immune checkpoint inhibitors can also be combined with drugs for synergetic therapy. A good example is the preparation of dual-pH responsive core–shell NPs by simultaneously presenting anti-PD-1 on the surface while encapsulating curcumin internally. The sequential release of anti-PD-1 and curcumin effectively enhanced the antitumor therapeutic effect.^55^ Tang et al. developed cell surface-conjugated nanogels that can load large quantities of protein drugs by a disulfide-containing cross-linker.^56^ When the nanogels are recognized by T cells, the reduction potential on the T cell surface increases and the disulfide bonds can be cleaved, resulting in the release of proteins *in situ*. By using an IL-15 superagonist complex, they expanded T cells 16-fold in tumors and increased the upper limit of cytokine administration. The improved therapeutic window could significantly increase tumor clearance *in vivo*.

**Figure 6:**
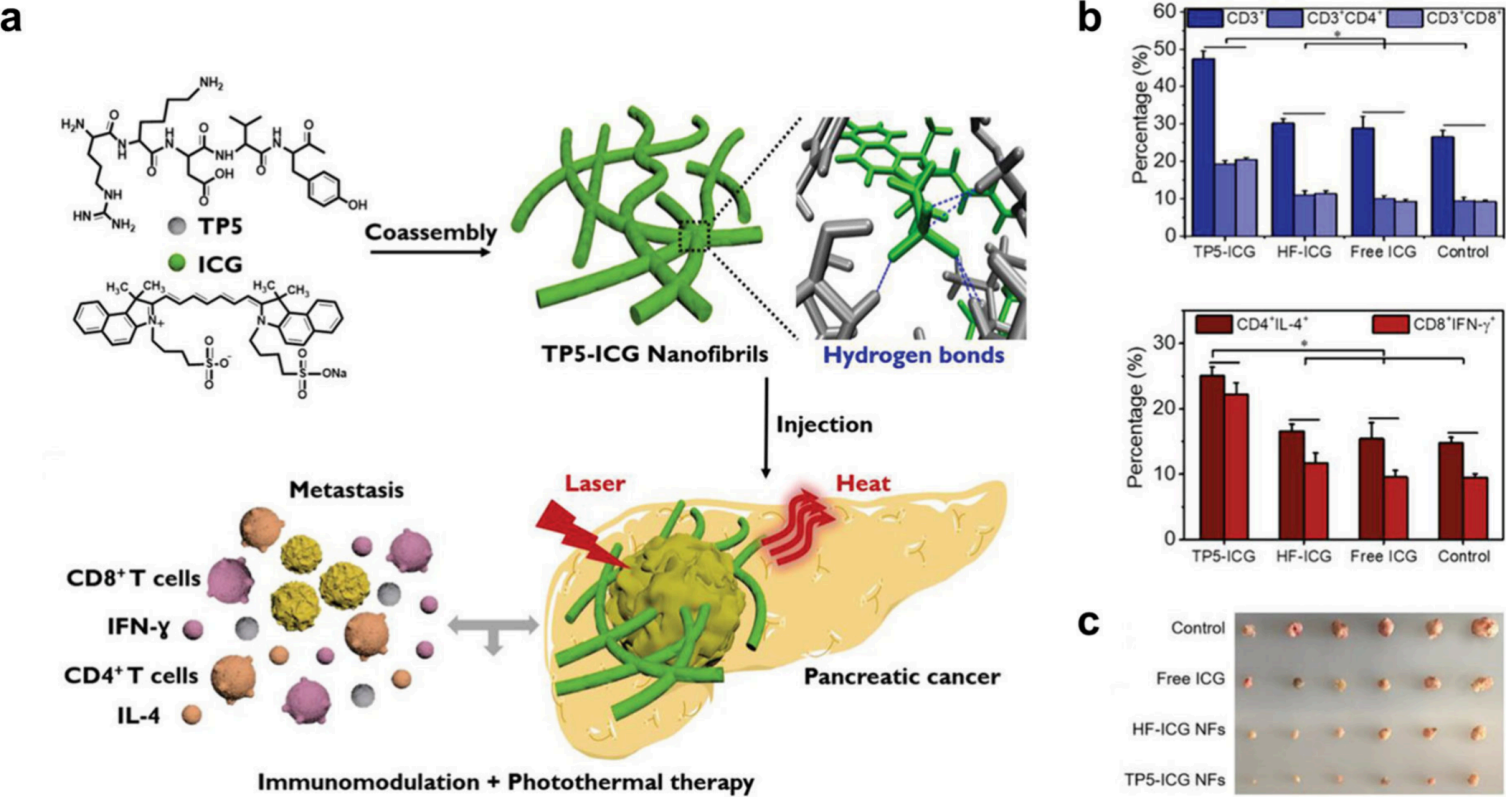
*In vivo* photothermal and immunomodulatory effects of TP5-ICG NFs. (**a**) Schematic illustration of the formation and application of the NFs. (**b**) Quantitative frequency of CD3^+^, CD3^+^CD4^+^, and CD3^+^CD8^+^ T cells (upper) and CD4^+^IL-4^+^ and CD8^+^IFN-γ^+^ T cells (lower) in mouse splenocytes after distinct treatments. (**c**) Photograph of tumors harvested from different groups of mice at 21 days. Reproduced with permission from ref (57). Copyright 2021 John Wiley and Sons.

Last but not least, combinational functionalization of NPs, that is, integrating other active molecules, such as photoactive drugs or tumor-associated/specific antigens (TAAs/TSAs), with immunologically active molecules, may synergistically enhance the therapeutic outcome in terms of chemo-immunotherapy and photo-immunotherapy. As a representative example, Yan and co-workers coassembled the clinically used photothermal agent indocyanine green (ICG) and pentapeptide thymopentin (RKDVY, abbreviated as TP5) to obtain nanofibrils (NFs) (Figure 6a).^57^ TP5 has been demonstrated to have immunomodulatory bioactivity including promoting thymocyte differentiation and modulating mature T cell function (Figure 6b). The hydrogen bonding occurring between the amide groups of TP5 and the sulfonate groups of ICG promotes the formation of long-range ordered NFs. In a refractory pancreatic tumor model, after one localized injection and one light irradiation, the rapid tumor ablation effect from ICG and mild systematic immunomodulation from TP5 potently inhibited the tumor growth and metastasis, while minimizing side effects (Figure 6c).

## 4 Conclusions and Outlook

Through immunomodulation, T cells can become more “active” and strengthen the body’s ability to fight infectious diseases and cancers. Today’s ACT exemplifies particle-assisted immunotherapy and has achieved remarkable clinical success in cancer treatment. Engineered T cells can be specifically expanded *ex vivo* in a robust manner with the assistance of MPs. However, the manufacturing process remains relatively lengthy and time-consuming, and achieving deep infiltration of engineered T cells within tumors after administration and avoiding exhaustion poses a significant challenge. Direct T cell modulation *in vivo* could offer a solution to this issue by bypassing *ex vivo* processes and improving therapeutic efficacy, representing a major future direction in this field. For example, it has been reported that potent CAR T cells can be generated *in vivo* by efficient delivery of modified mRNA via lipid NPs targeting T cells.^58^ Furthermore, nanoparticles with high mobility, known as nanomotors, have demonstrated their ability to enhance tumor infiltration of T cells *in vivo*.^59^

Immune checkpoint therapies present another promising strategy for treating cancer by blocking the negative inhibition of T cell function by cancer cells. The U.S. FDA has approved a total of six immune checkpoint inhibitors in three different categories, including PD-1, PD-L1, and CTLA-4 inhibitors. Particles have been shown to enhance the spatially and temporally controlled delivery of these antibodies, exhibiting better performance than free antibodies. Nevertheless, designing stimuli-responsive systems and applying them *in vivo* to mitigate side effects remains a significant challenge. In addition, combination therapies, such as utilizing BiTEs as a bridge directly connecting T cells and cancer cells, can serve as a flexible platform to regulate T cell responses for immunotherapy. The application of anchoring BiTEs to particles has not been extensively explored but is garnering increasing attention.

Besides these positive pathways, the treatment of autoimmunity is also a key aspect of immunomodulation. This can be achieved by inhibiting autoreactive T cells, reprogramming autoreactive T cells to T_reg_ cells, or activating T_reg_ cells. In all cases, particles have been shown to facilitate these processes by providing direct stimulation through contact with T cells or by delivering cytokines and immunosuppressive drugs. To achieve these goals, the further development of MPs and NPs should be prioritized to establish flexible regulatory platforms for T cells. Together with other immune cell therapies, such as DC-modulated cancer vaccines, we believe that therapeutic outcomes can better meet individual expectations in the future.

## Supporting Information Available

Introduction to the signals required for T cell activation, with Figure S1 showing a schematic of the signals delivered by APCs for full T cell activation (PDF)
