# Supplementary material for: Engineering Functional Particles to Modulate T Cell Responses
Source: Acc Mater Res. 2024 Jul 18;5(9):1048–58. doi: 10.1021/accountsmr.4c00105 (PMC11443481; doi:10.1021/accountsmr.4c00105)
Supplement: Supplementary file 1 — mr4c00105_si_001.pdf [file mr4c00105_si_001.pdf]

Supporting Information for

Engineering Functional Particles to Modulate T Cell Responses

Yudong Li,[‡ a] Shukun Li,[‡ a, b] Jari F. Scheerstra,[a] Tania Patiño,[a] Jan C. M. van Hest[*a] and Loai K. E. A. Abdelmohsen[*a]

[a] Bio-Organic Chemistry, Institute for Complex Molecular Systems, Eindhoven University of Technology, 5600 MB Eindhoven, The Netherlands

[b] State Key Laboratory of Biochemical Engineering, Institute of Process Engineering, Beijing 100190, China

‡ These authors contributed equally to this work and share first authorship

* Correspondence:

l.k.e.a.abdelmohsen@tue.nl

j.c.m.v.hest@tue.nl

Signals required for T cell activation

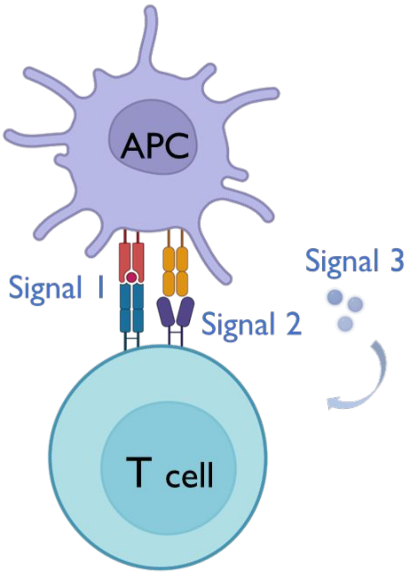

Figure S1. To fully activate T cells, antigen-presenting cells (APCs) deliver three necessary signals: 1) antigen presentation, 2) co-stimulation, and 3) cytokine release.

Signal 1: Antigen presentation. T cell activation is initiated upon the ligation of the T cell receptor (TCR) through a specific peptide-major histocompatibility complex (pMHC) presented by APCs, leading to the formation of the immunological synapse (IS). pMHC-I is specialized for $CD8^+$ T cell activation, while pMHC-II is specialized for $CD4^+$ T cell activation.[1] The activation can also be achieved when agonistic antibodies bind to the T cell's CD3 protein, which leads to the activation of the CD3 signal transduction pathway, essential for activation. This activation is however not antigen-specific.

Signal 2: Co-stimulation. In addition to antigen recognition by the TCR, a second co-stimulatory signal is required for optimal T cell activation and expansion. This co-stimulatory signal is provided by co-stimulatory molecules expressed on APCs. Receptors on T cells interact with these molecules, with CD28 being the most widely studied receptor. CD28 binds to the co-stimulatory molecules B7-1 (CD80) or B7-2 (CD86), which are necessary for T cell proliferation and cytokine production. Conversely, activation without the second signal may lead to T cell anergy or apoptosis.[2]

Signal 3: Cytokine release. After the initial activation by the first two signals, T cell immune responses, such as increased T cell survival and enhanced effector functions, can be further modulated by cytokines. For example, IL-12 and IFN-γ are essential for T_{h1}

development, IL-4 and IL-2 play a key role in T_{h2} differentiation, and TGF-β and IL-2 can induce the generation of T_{reg} cells.[3] In addition, cells can subsequently develop a responsive memory population.[4]

According to their functions, cytokines can be divided into pro-inflammatory cytokines and anti-inflammatory cytokines. Pro-inflammatory cytokines include IL-1α/β, IL-8, TNF-α, and IFN-γ, which primarily promote inflammation;[5] while anti-inflammatory cytokines include IL-4, IL-10, and TGF-β, which can inhibit inflammatory responses.[6] However, this classification is simplistic, and a cytokine may be a pro-inflammatory cytokine or an anti-inflammatory cytokine depending on the context.[7]

Signal 4: Nutrients. While the first three signals are considered critical for T cell activation, our understanding of T cell activity is evolving. Over the past two decades, the importance of metabolism in programming T cell function and differentiation has been increasingly recognized. Nutrients in the microenvironment, including glucose, amino acids, and lipids, can interact with the first three signals to influence the final outcome of the immune response. The latest understanding of Signal 4 and how it can be harnessed to improve the efficacy of immunomodulation can be found here.[8-11]

References

1. Rock, K. L.; Reits, E.; Neefjes, J., Present yourself! By MHC class I and MHC class II molecules. *Trends Immunol.* **2016,** *37* (11), 724-737.
2. Chen, L.; Flies, D. B., Molecular mechanisms of T cell co-stimulation and co-inhibition. *Nat. Rev. Immunol.* **2013,** *13* (4), 227-242.
3. Luckheeram, R. V.; Zhou, R.; Verma, A. D.; Xia, B., CD4+ T cells: differentiation and functions. *J. Immunol. Res.* **2012,** *2012* (1), 925135.
4. Curtsinger, J. M.; Mescher, M. F., Inflammatory cytokines as a third signal for T cell activation. *Curr. Opin. Immunol.* **2010,** *22* (3), 333-340.
5. Dinarello, C. A., Proinflammatory cytokines. *Chest* **2000,** *118* (2), 503-508.
6. Opal, S. M.; DePalo, V. A., Anti-inflammatory cytokines. *Chest* **2000,** *117* (4), 1162-1172.
7. Scheller, J.; Chalaris, A.; Schmidt-Arras, D.; Rose-John, S., The pro-and anti-inflammatory properties of the cytokine interleukin-6. *BBA - Mol. Cell Res.* **2011,** *1813* (5), 878-888.
8. Pearce, E. L., Metabolism in T cell activation and differentiation. *Curr. Opin. Immunol.* **2010,** *22* (3), 314-320.
9. Cohen, S.; Danzaki, K.; MacIver, N. J., Nutritional effects on T‐cell immunometabolism. *European journal of immunology* **2017,** *47* (2), 225-235.
10. Giles, J. R.; Globig, A.-M.; Kaech, S. M.; Wherry, E. J., CD8+ T cells in the cancer-immunity cycle. *Immunity* **2023,** *56* (10), 2231-2253.
11. Raynor, J. L.; Chi, H., Nutrients: Signal 4 in T cell immunity. *Journal of Experimental Medicine* **2024,** *221* (3), e20221839.
